# Critical Role of Synovial Tissue–Resident Macrophage and Fibroblast Subsets in the Persistence of Joint Inflammation

**DOI:** 10.3389/fimmu.2021.715894

**Published:** 2021-09-03

**Authors:** Samuel Kemble, Adam P. Croft

**Affiliations:** Rheumatology Research Group, Institute of Inflammation and Ageing (IIA), University of Birmingham, Queen Elizabeth Hospital, Birmingham, United Kingdom

**Keywords:** single cell transcriptomics, macrophage, fibroblasts, tissue resident cells, rheumatoid arthritis

## Abstract

Rheumatoid arthritis (RA) is a chronic prototypic immune-mediated inflammatory disease which is characterized by persistent synovial inflammation, leading to progressive joint destruction. Whilst the introduction of targeted biological drugs has led to a step change in the management of RA, 30-40% of patients do not respond adequately to these treatments, regardless of the mechanism of action of the drug used (ceiling of therapeutic response). In addition, many patients who acheive clinical remission, quickly relapse following the withdrawal of treatment. These observations suggest the existence of additional pathways of disease persistence that remain to be identified and targeted therapeutically. A major barrier for the identification of therapeutic targets and successful clinical translation is the limited understanding of the cellular mechanisms that operate within the synovial microenvironment to sustain joint inflammation. Recent insights into the heterogeneity of tissue resident synovial cells, including macropahges and fibroblasts has revealed distinct subsets of these cells that differentially regulate specific aspects of inflammatory joint pathology, paving the way for targeted interventions to specifically modulate the behaviour of these cells. In this review, we will discuss the phenotypic and functional heterogeneity of tissue resident synovial cells and how this cellular diversity contributes to joint inflammation. We discuss how critical interactions between tissue resident cell types regulate the disease state by establishing critical cellular checkpoints within the synovium designed to suppress inflammation and restore joint homeostasis. We propose that failure of these cellular checkpoints leads to the emergence of imprinted pathogenic fibroblast cell states that drive the persistence of joint inflammation. Finally, we discuss therapeutic strategies that could be employed to specifically target pathogenic subsets of fibroblasts in RA.

## Introduction

Rheumatoid arthritis (RA) is a chronic immune-mediated inflammatory disease (IMID) affecting approximately 1% of the UK population (1). Unlike osteoarthritis (OA) (a predominately non-inflammatory joint disease characterized by destruction of articular cartilage), RA is associated with systemic immune dysfunction resulting in persistent inflammation that localizes to synovial joints (2). If untreated, RA leads to irreversible joint damage and functional disability (3).

The introduction of biological, disease-modifying anti-rheumatic drugs (bDMARDs) has led to significant improvements in the treatment of RA. Current therapies target either specific immune cells, their secretory products or specific signaling pathways using small molecule inhibitors (4–9). The etiology and pathophysiology of RA is highly heterogeneous and despite advances in treatment, there is a ceiling of therapeutic response observed in which 30-40% of patients do not respond to currently available treatments regardless of the mechanism of action of the drug used (10). In addition, those individuals that achieve clinical remission often relapse once treatment is tapered or withdrawn (11, 12).

As more biological and targeted synthetic DMARDs are introduced into clinical practice, we are observing the emergence of a population of patients that have failed to respond to multiple drugs. This disease state is termed treatment refractory or difficult to treat RA (13, 14) and has recently been defined by a EULAR taskforce as the failure to respond to two bDMARDs of different mechanisms of action (15). However, the molecular and cellular basis of treatment refractory RA is yet to be determined and research strategies to address this area of unmet clinical need are urgently required (16). A key determinant of refractory RA is unidentified drivers of disease that modulate of the local synovial microenvironment (17–19). The failure to neutralize these pathways leads to disease persistence and inflammatory flare following treatment withdrawal. The next generation of therapeutics in RA will need to identify and target these cellular drivers of disease persistence, if we are to develop effective therapies for those individuals with treatment refractory RA (20).

Tissue resident cells such as synovial fibroblasts and macrophages form the underlying basis of the joint microenvironment and have been shown to contibue to tissue homeostasis, but also regulate the timing and duration of local inflammatory responses (21–26). Recent evidence suggests that such tissue resident cells are associated with significant phenotypic heterogeneity. These phenotypes are reflected at the transcriptional and functional level within distinct anatomical compartments of the synovial membrane determined by site-specific signaling cues (27–29). Whilst significant research has focused on the role of infiltrating leukocytes in the pathogenesis of RA, little prominence has been given to the cells which reside within the joint microenvironment.

Response to therapy is thought to depend on the “synovial signature” or tissue heterogeneity. In early RA, three major synovial pathotypes have been described with distinct underlying transcriptional signatures, including: lympho-myeloid (predominantly B cells with myeloid presence), diffuse-myeloid (predominantly myeloid without T and B cells) and pauci immune (30). Compared to other synovial pathotypes, treatment naïve lympho-myeloid patients have greater levels of disease activity, synovitis, immune cell infiltration and T and B cell activity and were more likely to require biological therapy at 12 month follow up (30, 31). Interestingly, anti-TNFα therapy was less effective in patients with a pauci-immune pathotype – a signature characterized by a prevalence of stromal cells (fibroblasts) (30). Thus, understanding the synovial pathology has the potential to stratify treatment interventions in the future (31, 32).

In this review, we will discuss the phenotypic and functional heterogeneity of tissue resident synovial cells in the context of joint homeostasis, inflammation and resolution. Furthermore, we will focus on important cross-talk which exist between different synovial tissue cells and finally, we will discuss potential new therapeutic strategies that target tissue resident synovial cells which could potentially restore homeostasis following chronic disease.

## Role of Tissue Resident Synovial Cells in Joint Homeostasis

The articular capsule is a bulbous structure that consists of a fibrous mesenchymal tissue termed the synovium which encapsulates all diarthrodial joints. The synovium forms a barrier around the synovial cavity containing synovial fluid (~1-2mL of highly viscous liquid) that is rich in hyaluronic acid and lubricin (encoded by the *HAS1* and *PGR4* gene respectively) (33–35). The function of synovial fluid is to maintain viscosity and elasticity of articular cartilage and to form a layer of lubrication to reduce friction between adjacent cartilage surfaces as well as adjacent surfaces of the synovium and cartilage (34). Within the synovial cavity, type I collagen and cartilage containing collagen type II, coat the articular surface of the bones (36).

The synovium is a connective tissue structure which contains two regions: a continuous, tightly packed lining or intima layer comprised of tissue resident fibroblasts and macrophages (1-3 cell layers thick), and an underlying connective tissue called the sub-lining layer or subintima which includes sparsely distributed tissue resident fibroblasts and macrophages, adipose cells and blood and lymphatic vessels (situated in deep regions) and minimal infiltrating inflammatory cells (35, 37). Under resting conditions, tissue resident fibroblasts and macrophages form a tightly organized immunological barrier isolating the joint cavity (37). As the joint cavity lacks associated blood vessels, leukocytes are believed to migrate from the sub-lining and through the lining layer barrier however, under resting conditions this process is highly regulated with very few immune cells present in the synovial fluid (33, 35).

The joint microenvironment is constantly exposed to minor trauma as a result of locomotion. To compensate, this dynamic tissue must be continuously re-modelled and repaired to maintain homeostasis. Recent evidence suggests, in RA, fibroblasts are transcriptionally heterogenous and have specialized functions depending on their anatomical location within the synovium (38–42). However, whether these specialized subsets of fibroblasts exist under resting conditions is yet to be fully elucidated. This is due to the challenges of acquiring healthy synovial biopsies, although the human joint cell atlas will soon provide an extensive characterization of the developing and normal musculoskeletal human tissues (43).

Given their location within the synovium, it is highly likely HAS1^+^ PRG4^+^ lining layer fibroblasts secrete hyaluronic acid and lubricin into the joint space, providing lubrication and nourishment to facilitate joint locomotion. *In vitro* evidence has also shown synovial fibroblasts exhibit the ability to control ion transport and maintain and re-model the joint architecture by secreting extra cellular matrix (ECM) components such as type II, IV, V and VI collagens, proteoglycans, fibronectin, laminin and tenascin, and proteases such as matrix metalloproteinases (MMPs) and cathepsins (44–47). Such processes permit nutrient exchange between the synovial fluid and the synovial membrane (48).

Tissue resident macrophages are distributed throughout the lining and sparsely in the sub-lining region of the healthy synovium and have been shown to play an essential role in normal tissue physiology (26, 27, 37). Historically, it was hypothesized that synovial macrophages derive from the bone marrow *via* circulating monocytes entering the joint tissue through blood vessels located within the sub-lining layer (49). However, it is now recognized that specialized tissue resident macrophages are seeded into tissues during embryonic development and can undergo self-renewal within the tissue (37).

Synovial tissue macrophages were previously identified by highly expressing CD68 and CD163 (50). From these populations, lining synovial macrophages were discriminated from sub-lining cells by their expression of Fc*γ*RIIIa suggesting that macrophages in the lining layer play an important role in cellular clearance (51, 52). However, contradictory findings (53–55) suggest these markers are inadequate to discriminate tissue resident from blood borne macrophages, prompting the discovery of alternative markers through the employment of single cell profiling technologies such as single cell RNA sequencing (scRNAseq).

Until recently, it was proposed the synovial lining layer surrounding the joint cavity consisted of an interwoven network of fibroblasts and macrophages. However, Culemann et al. (2019) (37) using advanced super-high resolution fluorescence microscopy in conjunction with scRNAseq transcriptomic profiling of mouse synovial macrophages, have revealed the lining layer consists of a highly organized population of epithelial-like (expressing tight junction proteins such as JAM1, ZO-1 & CLDN5 and genes associated with planar cell polarity *Fat4* and *Vangl2*) CX_3_CR1^+^ macrophages which form a physical (mediated by tight junctions) and immunological barrier between the synovial cavity and the synovial membrane. These lining macrophages sit directly adjacent to lining layer fibroblasts and express a gene signature related to an immune-regulatory phenotype expressing *Trem2* and Tam receptor genes such as *Axl* and *Mfge4*. Importantly, deletion of *Cx_3_cr1* expressing synovial macrophages caused barrier breakdown and uncontrolled joint inflammation. These studies challenge our previous understanding of the synovial microanatomy and function, instead emphasizing the primacy of the lining macrophage as a master regulator of joint homeostasis, functioning to suppress joint inflammation.

CX_3_CR1^+^ TREM2^+^ lining macrophages are long-lived with a lifespan of ~5 weeks. Under normal resting conditions this barrier is maintained through a pool of locally proliferating CX_3_CR1^-^ MHCII^High^ mononuclear cells that are embedded within the interstitial regions of the synovial sub-lining layer. In addition to CX_3_CR1^+^ TREM2^+^ macrophages, CX_3_CR1^-^ MHCII^High^ macrophages also give rise to CX_3_CR1^-^ RELMα+ expressing macrophages. These cells however are situated in the sub-lining and express CD206 and CD163. Macrophages expressing such markers have been shown to play an important role in M2 alternative activation supporting immune-regulation (56) and wound healing (57). In RA, this ultimately promotes the phagocytosis of apoptotic cells through *MERTK* expression (58). Collectively, these data suggest that under steady state conditions, immune-regulatory RELMα+ macrophages also support joint homeostasis.

**Figure 1:**
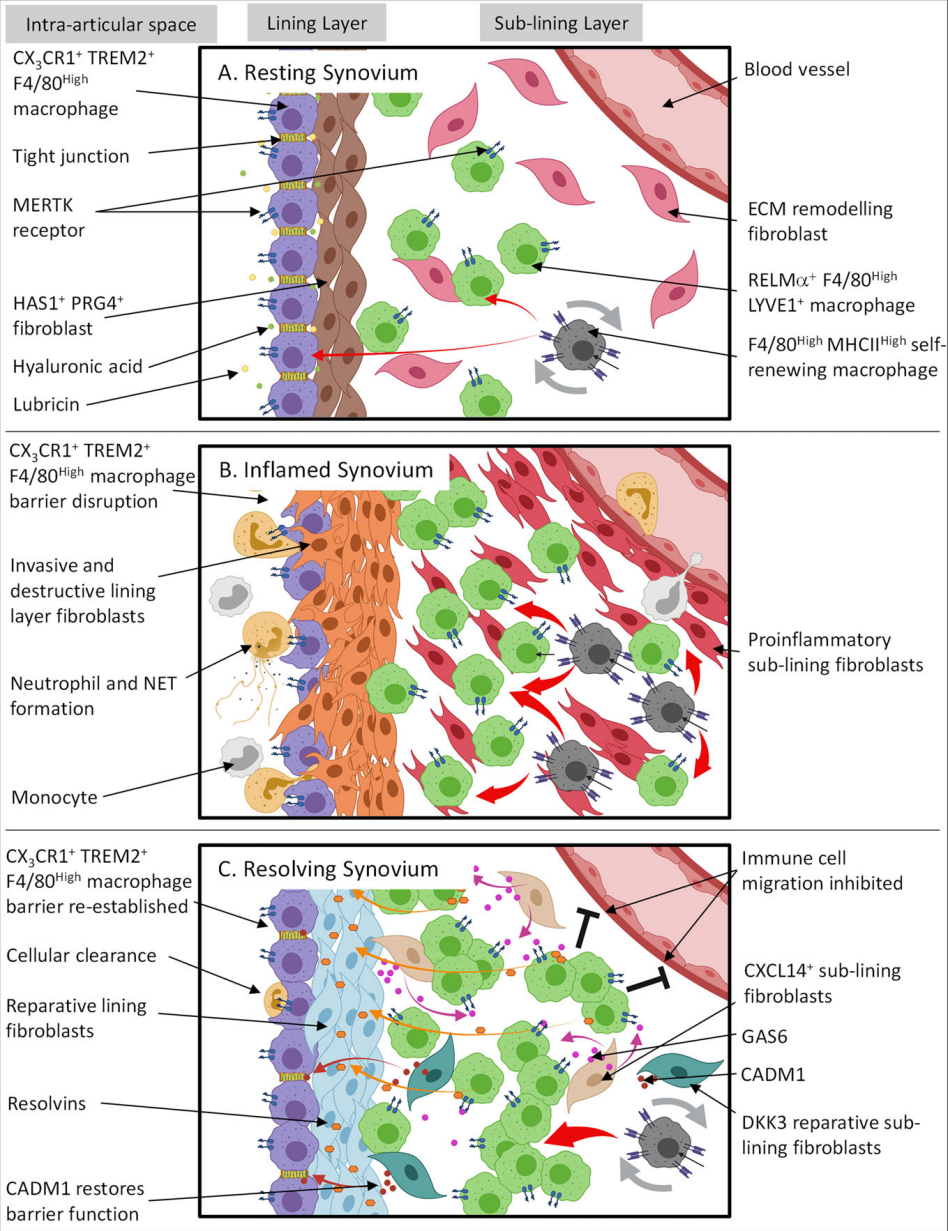
The spatial and temporal coupling between tissue resident synovial cells determines joint homeostasis, inflammation and resolution in RA. **(A)** Resting synovial tissue consists of both tissue resident macrophages and fibroblasts. Tissue macrophages contain an immune-regulatory population expressing MERTK and are repopulated by CX_3_CR1^-^ F4/80^High^ MHCII^High^ interstitial macrophages in the sub-lining. The lining layer is composed of highly organized, tight junction-mediated CX_3_CR1^+^ TREM2^+^ F4/80^High^ macrophages, forming an immunological barrier. Lining layer fibroblasts sit directly under CX_3_CR1^+^ TREM2^+^ F4/80^High^ barrier and secrete hyaluronic acid and lubricin into the intra-articular space *via* tight junctions to lubricate the joint cavity. The sub-lining is composed of sparsely distributed fibroblasts and RELMα^+^ LYVE1^+^ F4/80^High^ macrophages where the former is thought to constantly remodel ECM following mechanical trauma. **(B)** Although during arthritis the CX3CR1^+^ TREM2^+^ F4/80^High^ macrophage barrier becomes dysfunctional allowing migrating inflammatory myeloid cells and activated lining fibroblasts to invade the intra-articular space, these lining macrophages support resolution by retaining an immune-regulating phenotype and clearing apoptotic cells from the synovial cavity. RELMα^+^ LYVE1^+^ F4/80^High^ macrophages also expand in the sub-lining. However, it is unknown if this is to actively trigger resolution or drive inflammation. **(C)** For arthritis to resolve, cellular infiltration must cease. In conjunction, it is proposed that sub-lining CXCL14^+^ fibroblasts secrete GAS6 which interacts with MERTK on RELMa^+^ LYVE1^+^ F4/80^High^ macrophages causing the release of resolvins. Resolvins then switch lining fibroblasts from a proinflammatory to a reparative state to restore homeostasis. Also situated in the sub-lining are DKK3^+^ fibroblasts. The exact function of these cells is unknown; however, transcriptional evidence suggests they release proteins such as CADM1 which may promote the repair of CX_3_CR1^+^ TREM2^+^ F4/80^High^ macrophage barrier function. This figure is based on human and mouse data. Image created with (BioRender.com).

In agreement with Culemann et al. (37*)*, other studies in both mice and humans have shown under resting conditions two long-lived tissue resident macrophage subsets exist which express: in mice, F4/80^High^ MHCII^-^ and F4/80^High^ MHCII^+^ (59) and in humans, MERTK^+^ TREM2^+^ lining and MERTK^+^ LVYE1^+^ sub-lining macrophages (60). It is possible these populations are analogous to CX_3_CR1^+^ TREM2^+^ and CX_3_CR1^-^ RELMα^+^ macrophages, respectively. See diagram in **Figure 1A** for a summary of the role in which tissue resident synovial cells play in joint homeostasis.

In summary, tissue resident synovial cells not only provide the architecture of the joint but also play an important functional role in joint homeostasis. Heterogeneity has been reported within synovial macrophage and fibroblast subsets and this appears to be dependent on anatomical location within the joint microenvironment. Although these subsets of synovial cells differ in their transcriptomic signatures, the functional characteristics of these subsets under resting conditions remains to be fully elucidated.

## Persistent Joint Inflammation in RA Renders Normal Joint Homeostasis Ineffective

During established RA, the synovium becomes hyperplasic with an increase in cellularity, resulting in an expansion of the synovial membrane between 10-20 cell layers thick. In the sub-lining, extensive angiogenesis occurs supporting a mass influx of infiltrating immune cells, such as lymphocytes, plasma cells and monocyte-derived macrophages (3, 61). For the purpose of this review, the role in which infiltrating cells play in RA [reviewed in (5, 62)] will not be discussed further. The hyperplasic synovium contains a heterogeneous population of tissue resident fibroblasts and macrophages that play differing roles in regulating and promoting joint inflammation and damage (63–68). While synovial macrophage numbers have been shown to positively correlate with disease severity (69, 70), synovial hyperplasia is predominantly a result of increases in fibroblast cell numbers thought to be due to proliferation (71), increased survival (72) and potentially migration (73).

In chronically inflamed joints the hypertrophied synovium forms pannus tissue (74) that adheres and degrades the articular cartilage (the cartilage-pannus junction) as a result of fibroblast secreted proteases (75). These proteases [such as matrix metalloproteinases (MMPs)] activate chondrocytes which in turn secrete additional proteases forming a positive endogenous feedback loop (76). For pannus formation to occur, the synovium must be re-organized into selectively expanded regions which support immune or non-immune processes (48). This re-modeling of the synovial membrane into distinct microenvironmental niches is thought to be the result of ECM remodeling by tissue fibroblasts. For example, Cadherin-11 (CDH11) is an adhesion molecule expressed by synovial fibroblasts during RA (77) and is critical for synovial hyperplasia (78). The genetic deletion of *Cdh11* impairs pannus formation and attenuates inflammation *in vivo (*78, 79). Thus, formation of hyperplasic synovial tissue is essential for supporting joint inflammation and pathogenic fibroblast behaviour in arthritis.

Fibroblast migration may also be an important contributor to fibroblast expansion in the joint and thus synovial hyperplasia. Mouse synovial fibroblasts exhibit the ability to transmigrate *in vivo* through blood vessels and spread arthritis to unaffected joints, through mesenchymal precursors (80). These fibroblasts were found to be indispensable for inducing onset of arthritis in non-inflamed joints suggesting they may be directly arthrogenic (81). Fibroblasts implanted into a cartilage/sponge matrix in an immune deficient mouse also exhibit the ability to migrate from the engraftment site to a distant cartilage implant (82). Work from our group has shown these migrating fibroblasts express podoplanin (PDPN) and not sub-lining fibroblast markers such as CD248 (38). However, as migration progressed, the synovial tissue architecture developed both lining and sub-lining regions suggesting to some extent fibroblasts are plastic and respond to micro-environmental cues.

Recent evidence in humans, supports the notion fibroblasts might migrate to non-inflamed tissue through pre-inflammatory mesenchymal cells (PRIME cells) (73). PRIME cells are detectable in human blood prior to arthritis flare and are therefore a potential biomarker for predicting relapse in RA. It is hypothesized that PRIME cells are recruited by naïve B cells, a process which is reduced following the onset of joint symptoms. Transcriptomic analysis shows that PRIME cells may represent an analogous gene signature to proinflammatory fibroblasts identified in the sub-lining layer (42). Further work is required to determine whether PRIME cells truly migrate into the joint and if so, the mechanism by which these cells induce local disease flare within the joint itself.

Although pannus formation is hypothesized to be predominantly a fibroblast mediated process, key alterations of the synovial structure are initially driven by a failure of the synovial macrophage barrier. Using 3D light-sheet fluorescence microscopy, Culemann et al. (37), showed immune complex mediated synovial lining barrier dysfunction occurs in response to serum transfer induced arthritis (STIA) (37). Although these CX_3_CR1^+^ TREM2^+^ lining macrophages remained in the same position they changed orientation and morphology and permitted proliferating fibroblasts to invade the lining space and neutrophils and monocytes to migrate into the intra-articular space (**Figure 1B**). Although the fraction of CX_3_CR1^+^ TREM2^+^ macrophages did not expand in response to different types of experimental arthritis, they did maintain an immune-regulatory phenotype expressing Tam receptors such as Axl and Mfge4, clearing apoptotic cell bodies from the synovial space. In conjunction, sub-lining CX_3_CR1^-^ RELMα^+^ macrophages expand.

We propose that CX_3_CR1^+^ TREM2^+^ lining macrophages can be considered as the first cellular checkpoint in the synovium that attempts to suppress joint inflammation and restore normal joint homeostasis following an inflammatory challenge. The functional purpose of the spatial coupling between lining layer macrophages and lining fibroblasts (which are functionally, spatially and anatomically distinct from sub-lining fibroblasts) is currently unknown, but we hypothesize that the loss of this spatial coupling during inflammation may lead to the emergence of a compensatory repair like phenotype in lining fibroblasts, however this remains to be definitely proven.

Lining CX_3_CR1^+^ TREM2^+^ and sub-lining CX_3_CR1^-^ RELMα+ macrophages differentiate from tissue resident, self-renewing CX_3_CR1^-^ MHCII^High^ interstitial macrophages (37). Under resting conditions, CX_3_CR1^-^ RELMα^+^ macrophages express genes associated with alternative activation, suggesting an immunosuppressive phenotype that is protective. However, the expansion of this population of macrophages in arthritis, suggests that either there is a compensatory response in which these macrophages attempt to suppress inflammation and reinstate homeostasis or unknown signals from within the tissue switch their phenotype to a proinflammatory cell state. The latter is controversial as unlike highly plastic monocyte-derived macrophages, it is hypothesized resident macrophages are less plastic (83). However, investigators combining fate mapping, scRNAseq and epigenetic studies and investigating the role of macrophage subsets in the pathogenesis of pulmonary fibrosis have suggested plastic monocyte-derived macrophages can differentiate into tissue resident macrophages, giving rise to inflammation-imprinted, tissue resident macrophages which drive and sustain an inflammatory response (83).

In addition to the above, synovial fibroblasts are known to modulate macrophage gene expression profiles (84) and therefore, could determine macrophage phenotype in RA. In support of this, a recent study showed scRNAseq transcriptomics of CD14+ macrophages in the RA synovium revealed a unique expanded subset of HBEGF+ macrophages and when co-cultured with TNFα-activated synovial fibroblasts these macrophages were transcriptionally modified (85). This change in polarization was shown to be similar to classically activated pro-inflammatory macrophages, however as the HBEGF+ macrophage phenotype was dependent on fibroblast-derived prostaglandin_2_ (PGE_2_) it was considered distinct from classical or alternative polarization and altered the way these macrophages could respond to standard treatments used in RA. These inflammatory macrophages were able to promote fibroblast invasiveness in an epidermal growth factor dependent manner. It is therefore possible that they play a role in determining the success or failure of the immune-regulatory TREM2^+^ CX_3_CR1^+^ macrophage barrier. Also, fibroblast-derived mediators such as PGE_2_ may alter the transcriptional profile of CX_3_CR1^-^ RELMα^+^ sub-lining macrophages favoring a proinflammatory phenotype in response to the inflammatory joint milieu in RA. These concepts have yet to be fully tested.

To answer the above questions, it is important to understand the mechanism in which CX_3_CR1^-^ MHCII^High^ macrophages give rise to anatomically distinct synovial subsets under normal and perturbed states and how interactions with fibroblasts alter these immunophenotypes. Is there a pool of CX_3_CR1^-^ MHCII^High^ macrophages which is primed but readily depleted in response to an inflammatory stimulus? If CX_3_CR1^-^ RELMα^+^ macrophages are proinflammatory, what might switch these cells from an immunosuppressive to proinflammatory state? These are important questions that remain to be answered.

## Tissue Resident Synovial Cells Re-Establish Joint Homeostasis in RA Patients

The recent identification of distinct tissue resident synovial macrophage (37) and fibroblast (39–42, 86) subsets in various disease states in RA, suggests the function of these cells is dependent on both their anatomical location within the synovium and the tissue disease state. Furthermore, it appears that tissue resident macrophage phenotype may be more stable than the fibroblast state. Thus, dysregulated association between these two tissue resident cell types may not only determine the timing of inflammation but also its duration.

Another possible cellular immune checkpoint may exist between type I tyrosine kinase receptor (members of the TAM family) expressing synovial macrophages and sub-lining fibroblast subsets through the release of endogenously produced GAS6 (60). Macrophage type I tyrosine kinase receptors consist of TYRO3, AXL and MER. The role in which these receptors may play in regulating joint inflammation in arthritis was first demonstrated in *Tam* deficient mice where spontaneous arthritis (amongst other inflammatory manifestations) was reported (87). In agreement with Culemann et al. (2019) (37), an investigation in humans by Alivernini et al. (2020) (60) has shown two general subsets of synovial macrophages (tissue resident MERTK^+^ and infiltrating MERTK^-^) with differential enrichment of these populations within the synovium depending on tissue stage where risk of disease flare was determined by the ratio of these two subpopulations in the synovium.

The above authors investigated synovial biopsy tissue from healthy individuals, patients with active RA and those individuals in sustained clinical remission under methotrexate combined with TNF inhibition (60). Monocyte-derived MERTK^-^ macrophages were expanded during active RA and identified as proinflammatory, expressing either alarmins (S100A12 and IL-1β), bone remodelling proteins (CD48 and SPP1), interferons (HLA and ISG15) or molecules related to antigen presentation (HLA and CLEC10A). In contrast, tissue resident MERTK^+^ macrophages were enriched in healthy synovial tissue and synovial tissue from those individuals in clinical remission. As others have shown *Mertk-/-* mice exhibit exacerbated arthritis in response to STIA (88), This suggests, a dysregulation in macrophage TAM signaling appears to determine autoimmunity and drive chronic inflammation in RA.

Immune-regulatory MERTK^+^ macrophages consisted of high expression of either TREM2 or LYVE1 demarking their lining or sub-lining location, respectively (60). Culemann et al. (2019) also reported CX_3_CR1+ lining macrophages express TREM2 (37). *Trem2* expression has proven to inhibit inflammation in mouse microglia by suppressing NF-κB signaling (89). In contrast, LYVE1+ macrophages have been shown to engage with hyaluronan on smooth muscle cells and induced MMP-9 dependent degradation of collagen to prevent its deposition and therefore vascular stiffness (90). This suggests, under resting and resolving states, that MERTK^+^ macrophage subsets may not only regulate inflammation but potentially also fibroblast collagen production and tissue stiffness of the joint.

Endogenous pro-resolving mediators such as resolvins are cardinal biomarkers and active regulators of resolution in RA and are now considered a potentially useful biomarker of DMARD therapy (91). *In vitro* functional assays performed by Alivernini et al. (2020) showed MERTK^+^ macrophages released resolvin D1 and induced a reparative phenotype in lining fibroblasts (increasing the expression of collagen genes such as *COL1A*) driven by GAS6 secretion from a sub-lining fibroblast subset expressing THY1 and CXCL14 (**Figure 1C**). GAS6 acts as a bridging molecule to promote the binding of MERTK with exposed phosphatidylserine (PS) on apoptotic cells and induces phagocytosis (92, 93). This anti-inflammatory/pro-resolving process ultimately causes suppression of NFκB (94). Furthermore, cleavage of the ectodomain of MERTK by ADAM metallopeptidase domain 17 (ADAM17) has been shown to exacerbate tissue inflammation by limiting the production of specialized pro-resolving mediators (95, 96).

The MERTK/GAS6 axis has been shown to suppress proinflammatory cytokine production (97) and induce IL-10 production by alternatively activating anti-inflammatory macrophages (98). Importantly, GAS6 production by synovial fibroblasts and within synovial fluid is reduced in RA joint and increased in the synovium of patients in sustained remission (60, 99, 100). Together these findings suggest diminished MERTK/GAS6 association is essential for resolution of RA and re-establishing joint homeostasis. In contrast, the loss of the MERTK/GAS6 axis following persistent proinflammatory stimuli might contribute to chronic inflammation.

Intriguingly, the transcriptional cassettes of MERTK^+^ macrophages from healthy synovial tissue and the synovial tissue of those individuals with RA in sustained clinical remission were reported to differ (60). MERTK^+^ macrophages in patients in stable remission expressed genes which encode Kruppel-like factor 4 (KLF4) and nuclear receptor subfamily 4 group a member 2 (NR4A2). Historical evidence shows KLF4 is expressed in the differentiating layers of the epidermis and has been shown to play an important role in establishing barrier function (101). Furthermore, both of these transcription factors have been shown to be regulators of macrophage polarization where mice lacking macrophage- or myeloid-specific KLF4 expression demonstrate increased proinflammatory characteristics and delayed wound healing (102), whereas NR4A2 (a target of macrophage migration inhibitory factor (MIF) signaling) expression has proven to curb inflammation and promote an anti-inflammatory phenotype by reducing IL-6 production (103). More recently, a study demonstrates KLF4 and KLF2 expression in macrophages regulates immune cell apoptosis as well as suppressing toll-like receptor (TLR) responses to nucleic acids to ensure maintenance of homeostasis (104).

In addition to immune-regulatory tissue resident macrophages, sub-lining fibroblasts expressing markers such as dickkopf WNT signaling pathway inhibitor 3 (DKK3), osteoglycin (OGN), cell adhesion molecule 1 (CADM1) & microfibrillar-associated protein 2 (MFAP2) are present in inflamed murine and RA synovium (39, 42). *DKK3* encodes DickKopf3 and overexpression of this protein in keloid fibroblasts has been shown to suppress cell proliferation and promote apoptosis *via* TGF-β1/SMAD signaling (105) as well as impairing tumor growth by promoting IL-7 production (106). Interestingly, *DKK3* expression is upregulated in OA and inhibits cartilage degradation *in vitro (*107). In contrast, OGN supports ectopic bone formation and its expression increases in the RA synovium in response to low-level laser therapy (108). The relationship between the fraction of DKK3+ fibroblasts and RA pathology, duration of disease and tendency of patients to remain in clinical remission is yet to be explored however, it is possible that DKK3+ fibroblasts could play a role in promoting resolution by supporting the re-establishment of joint homeostasis through modulation of joint repair pathways. Furthermore, DKK3+ fibroblasts also express CADM1 and MFAP2 which have been shown to be involved in enhancing intestinal barrier function in rats with inflammatory bowel disease (109) and fibroblast-mediated elastic fiber formation (110), respectively. As there is evidence suggesting DKK3+ embryonic cell lineages remain in joint tissue at maturity (111) it is possible DKK3+ fibroblasts have a suppressive role in healthy joint tissue supporting the synovial macrophage barrier, but this is yet to be investigated. The role tissue resident synovial cells play in resolution of inflammation and restoration of joint homeostasis is summarized in **Figure 1C**.

In summary, these studies demonstrate that both tissue resident macrophages and fibroblasts play an important role in supporting the resolution of joint inflammation in RA. The coupling of these cell types and outcome of their cross-talk appears to be critical in determining whether inflammation persists or resolves. We propose that these tissue resident cells form critical cellular checkpoints within the synovium that attempt to regulate the local inflammatory response. In this way macrophages can act as critical off switches by suppressing the phenotype of pathogenic fibroblasts. As a result, the maintenance of these cellular checkpoints facilitates resolution of joint inflammation and maintains disease remission, whereas their failure leads to the persistence of joint inflammation. The reasons such cellular checkpoints could fail include either quantitative changes to the proportions of the cellular subsets within the tissue that make up these checkpoints or the failure of the inter-cellular communication pathways between the two cell types. It is probable that persistently activated fibroblasts in chronic disease undergo epigenetic modifications which give rise to pathogenic subsets and ultimately render tissue resident stroma-macrophage checkpoints ineffective. As macrophages and fibroblasts appear to play an important role in homeostasis and disease progression, we will need to focus on understanding how these cells communicate in the context of inflammation and resolution. This will unravel the functional networks established by these tissue resident synovial cells at different stages of disease and in response to treatment, allowing us to develop novel therapeutic approaches to treat refractory RA and restore joint homeostasis resulting in sustained clinical remission.

## Pathologic Synovial Fibroblasts as Cellular Drivers of Treatment Refractory RA

Fibroblasts provide the architectural and functional landscape for tissue microenvironments (21, 22). In arthritis, synovial fibroblasts amplify inflammation and tissue damage driving multiple aspects of pathology including production of chemokines, cytokines and proteases which affect the recruitment, retention and differentiation of infiltrating leukocytes as well as the erosion of cartilage and bone (19, 46, 63, 112–114).

Historical studies regarding synovial fibroblast heterogeneity in RA have focused mainly on tissue topography, positional identity, surface protein expression of candidate cell markers and identifying the lining and sub-lining layers as distinct anatomical regions (79, 115–117). Recent efforts through the use of scRNAseq transcriptomics have defined the cellular diversity at the transcriptional level and research is ongoing to link this heterogeneity to cellular function and role in disease pathology (39–42). The first study to utilize this strategy was by Stephenson et al. (2018) (41) and showed two transcriptionally distinct human fibroblast subsets in RA which were demarcated by anatomical location – *CD55^+^* lining and *THY1^+^* (*CD90^+^*) sub-lining fibroblasts. Bulk RNA sequencing of these two populations with gene-set enrichment analysis, confirmed *CD55^+^* lining fibroblasts highly expressed *HAS1* whereas *THY1^+^* sub-lining fibroblasts expressed genes associated with ECM degradation and remodeling. *THY1*^+^ fibroblasts could be further defined by *PDPN^+/^-* populations where the former was considered a perivascular marker suggesting *THY1^+^* fibroblasts associate with blood vessels in the sub-lining.

Since Stephenson et al. (41), multiple researchers have attempted to unravel the phenotypic difference between fibroblast subsets in RA using sort purified, enzymatically digested human and mouse synovial tissue samples (39, 40, 42, 86). For example, Mizoguchi et al. (2019) (40) sorted synovial fibroblasts from human RA biopsies using markers which are upregulated in active RA synovium: PDPN, CDH11, CD34 and THY1 (38, 79, 116, 118). Using microarray analysis with bulk sequencing and principle component analysis, three unique PDPN+ fibroblast gene signatures were identified: 1) *CD34^-^ THY1^-^* lining layer fibroblasts expressing *MMP1*, *MMP3*, *PRG4* and *HAS1*; 2) *CD34^+^ THY1^+^* perivascular fibroblasts associated with sub-lining vasculature and 3) *CD34^+^ THY1^-^* distributed in superficial lining layer and throughout deep sub-lining. *CDH11* expression was observed in most fibroblasts with the greatest expression in the lining subset matching previous findings that *CDH11* expression promotes invasive and destructive behavior (79). Interestingly, of these populations, the *CD34^+^ THY1^+^* perivascular subset was expanded in RA and not OA and correlated with leukocyte infiltration and histological scores of synovitis and hypertrophy, whereas *CD34^-^ THY1^-^* lining were expanded in OA and not RA, suggesting synovial fibroblast subset expansion is dependent on disease type and context.

Expansion of pathological fibroblast subsets in the sub-lining of RA synovial tissue has also been demonstrated by others (39, 40, 42, 86). Zhang et al. (2019) integrated scRNAseq transcriptomics and mass cytometry of RA synovial biopsies and based on *CD45^-^ CD31^-^ PDPN^+^* expression identified three *THY1^+^* sub-lining fibroblasts subsets: *CD34^+^*, *HLA-DR^High^* and a unique subset expressing *DKK3^+^ (*42). Of the sub-lining subsets the *HLA-DR^High^* and *CD34^+^* populations were expanded in leukocyte-rich RA synovial tissue where the former was as high as a 15-fold increase. In agreement with Mizoguchi et al. (2019) (40) lining layer fibroblast in this study were also determined by *CD34^-^ THY1^-^ PRG4^+^* as well as *CD55^+^*.

Findings from our group show in the synovium of acute and chronic experimental arthritis models in mice and human RA synovial biopsy tissue, fibroblasts significantly increase expression of FAPα (fibroblast activation protein alpha), a cell membrane dipeptidyl peptide which was absent under resting conditions (39, 117). This led to the hypothesis that FAPα is a marker of pathogenic fibroblasts subsets in inflammatory disease. Deletion of FAPα expressing fibroblasts in mice not only suppressed inflammation but also reduced tissue damage thus confirming its pathogenic role in arthritis. FAPα fibroblasts could be broadly separated into THY1^-^ and THY1^+^ clusters reflective of their anatomical location in the joint synovium: THY1^-^ lining and THY1^+^ sub-lining layer fibroblasts. Furthermore, scRNAseq demonstrated THY1^-^ fibroblasts contained a lining layer gene signature expressing *Cd55*, *Prg4* and Clic5, whereas a THY1^+^ sub-lining signature expressed other markers such as *Cd34* and *Clec3b*. When comparing our mouse THY1+ scRNAseq data with human subsets identified by Zhang et al. (2019) (42), we found three transcriptionally analogous fibroblasts subsets: 1) *CD34^+^ C3^+^ MFAP5^+^* associated with blood vessels in the sub-lining perivascular space, expressing genes related to immuno-inflammatory processes and stromal memory and 2) *COL1A1^+^ COL8A1^+^ MDK^+^* expressing genes involved in bone, cartilage and ECM re-modelling. In addition, a single THY1^-^ lining subset was identified across species containing a transcriptional profile of *CLIC5^+^*, *TSPAN15^+^* and *PRG4^+^*. Importantly, re-analysis of Zhang et al. (2019) (42) data confirmed pathogenic *HLA-DR^High^* fibroblasts were also positive for FAPα.

**Figure 2:**
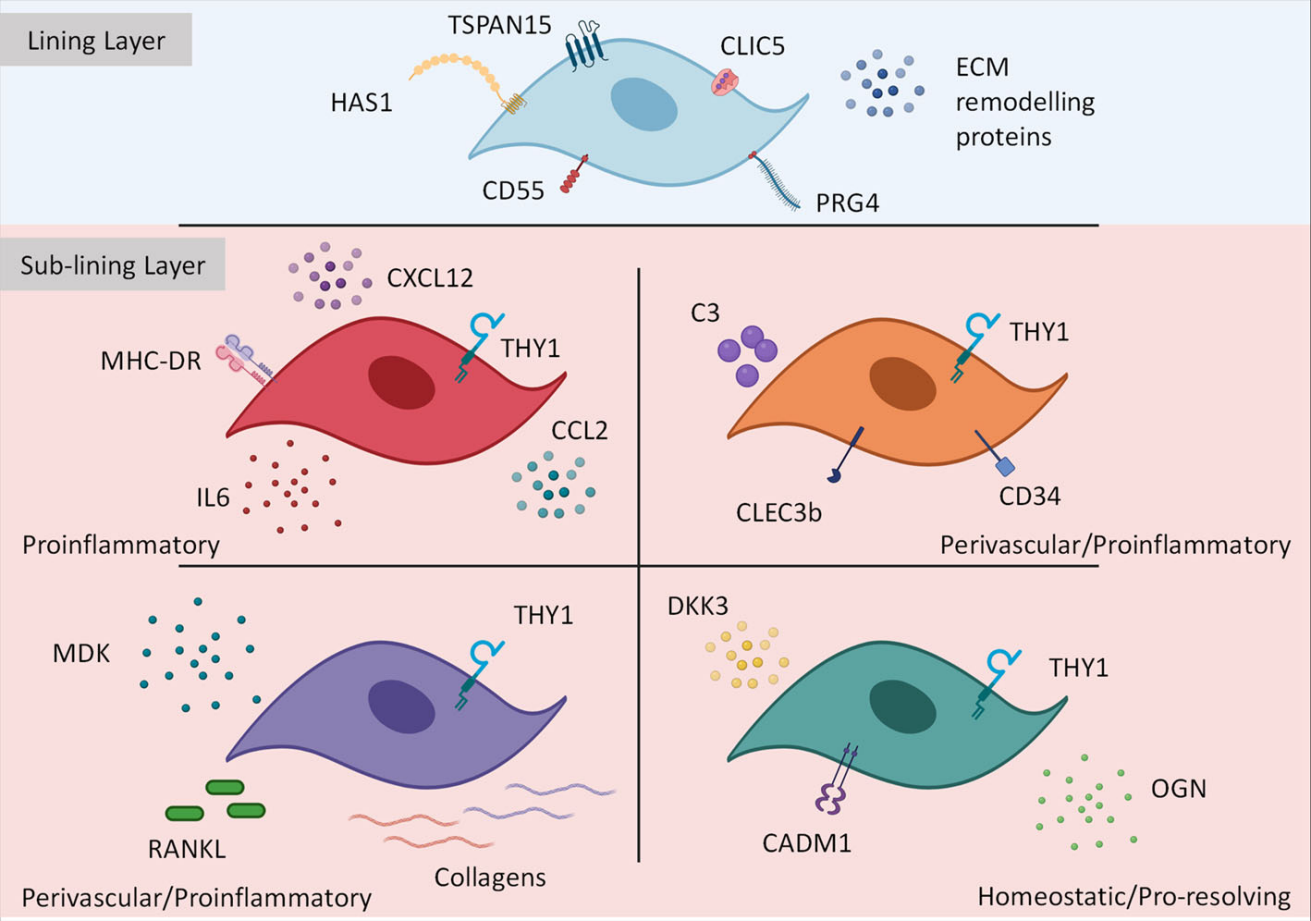
Transcriptionally and functionally distinct synovial fibroblast subsets within the inflamed joint. Synovial fibroblasts exist as distinct subsets or activation states. Lining layer fibroblasts play an important role in tissue repair and remodelling however, in response to inflammation these fibroblasts may play a role in bone and cartilage destruction. In contrast, sub-lining fibroblasts discriminated by *THY1* expression (with a transcriptional gradient extending from the vascular loci (highest *THY1* expression) to the synovial lining) expand during inflammation Within the sub-lining tissue, four differentially activated fibroblast subsets have been identified expressing distinct transcriptional cassettes determined by anatomical location within the joint. This figure is based on human and mouse data. Image created with (BioRender.com).

From the above studies it is clear FAPα^+^ THY1^+^ sub-lining fibroblasts contain distinct fibroblast subsets with non-overlapping effector functions. For example, *THY1^+^ CD34^+^* fibroblasts reported in all studies were shown to cluster in the perivascular region of the sub-lining tissue and from gene-set enrichment analysis and *in vitro* assays were shown to express inflammatory mediators such as IL-6, CXCL12 and CCL2 with the potential to facilitate immune cell infiltration. These subsets also express complement C3 and are responsible for stromal memory and tissue priming (39, 40, 42). Therefore, these populations can be generally grouped into *THY1^+^ CD34^+^ C3^+^* perivascular proinflammatory fibroblasts and importantly their expansion in RA has been shown to correlate with histopathological observations including synovial hypertrophy and inflammation (40). Recent work has shown that following repeated inflammatory stimuli, *CD34^+^ C3^+^* synovial fibroblasts become metabolically rewired and express high amounts of complement proteins to support local, inflammation driven tissue priming (113). This robust tissue response could define the switch from resolving to chronic joint inflammation observed in RA. In contrast, *CD34^-^* fibroblasts can be separated into two populations: *HLA-DR^High^* comprised of a similar proinflammatory phenotype as above as well as expressing genes associated with antigen presentation and IFN*γ* signaling (42), and *RANKL^High^* expressing fibroblasts which also express low osteoprotegerin (OPG) and exhibit the ability to drive osteoclast differentiation *in vitro (*40). An enhanced migratory and invasive behavior has been reported in this subset following exposure to platelet derived growth factor BB (PDGFBB) in a trans-well matrix invasion assay (40), therefore these cells may play an important role in cartilage and bone destruction. In addition to sub-lining fibroblasts, work from our group and others has shown that lining FAPα^+^ THY1^-^ fibroblasts also express genes that associate with osteoclast formation and activity (CCL9, RANKL) as well as cartilage degradation (MMP3, MMP9 and MMP13) consistent with a role in mediating bone and cartilage destruction (39, 40). See **Figure 2** for summary of pathological fibroblast subsets in rheumatoid arthritis.

To confirm differences in RA fibroblast effector functions *in vivo*, our group adoptively transferred FAPα^+^ THY1^+^ sub-lining and FAPα^+^ THY1^-^ lining fibroblasts into the joint of STIA and collagen-induced arthritis (CIA) mice and demonstrated THY1^+^ cells exacerbated disease by augmenting proinflammatory cytokine and chemokine production and promoting cellular infiltration. In contrast THY1^-^ cells had no effect on inflammation *in vivo* but did enhance bone and cartilage destruction both *in vivo* and *in vitro* (39). Such findings enhance our understanding how fibroblast subsets drive chronic inflammation. Understanding how fibroblast subsets relate to the global synovial tissue pathotype and how changes in their phenotype or quantitative proportions underlie specific disease endotypes such as treatment refractory RA will be an important area of future research.

## Endothelial-Derived Notch Signaling Induces Pathogenic Fibroblast Expansion in Arthritis

It remains unclear whether fibroblast heterogeneity is due to developmental determinants, cellular differentiation and/or epigenetic modifications. Recent studies have demonstrated differences in the expression of homeobox (*HOX*) family of transcription factors and WNT signaling pathways between fibroblasts isolated from different joints (119). The *HOX* gene signature shared similar features to the embryonic positional *HOX* gene expression pattern along the proximal–distal and anterior–posterior developmental axes. Others have shown that epigenetic modifications such as DNA methylation and histone modifications determine *HOX* gene expression in synovial fibroblasts (120, 121). It is thought that epigenetic imprinting of fibroblasts within a specific joint exists to support the unique biomechanical features of that joint. This anatomical diversity of synovial fibroblasts, while not disease specific, may underpin the tissue tropism observed in RA with positionally imprinted risk signatures of synovial fibroblasts determining the specific pattern of joint involvement in disease (122).

Synovial fibroblasts are also highly responsive to changes in the joint microenvironment with a phenotype dependent on microenvironmental instructive cues (86). In support of this, a study by Wei et al. (2020) (86) used *THY1* and *PRG4* as canonical markers of lining and sub-lining layer fibroblasts to anatomically define these regions of the synovial microanatomy and demonstrated a transcriptional *THY1/PRG4* gradient in RA synovial tissue. This suggests transcriptional intermediate fibroblast states exist between these two anatomical poles with the anatomical endpoint of the sub-lining expression signature being the mural cells (vascular smooth muscle cells and pericytes that surround microvasculature) which corresponded with the site of highest THY1 expression. Importantly, similar to Mizoguchi et al. (2018) (40), these transcriptional signatures were dependent on their positional location within the synovial tissue and were lost following ex-vivo expansion in culture. These findings collectively suggest that fibroblasts are passive responders to the joint microenvironment and their transcriptional phenotype is determined by their position in the tissue including their distance from the synovial vasculature.

Wei et al. (2020) (86) showed that THY1^high^ fibroblasts share similar anatomical sites as endothelial cell (EC) and mural cells. When cultured with or without EC in a free-floating 3D synovial tissue organoid system, fibroblasts form a lining and sub-lining layer in both conditions but only displayed a sub-lining transcriptional phenotype in the presence of EC. In the absence of EC, the fibroblast transcriptional signature became null where all fibroblasts expressed an intermediate transcriptional gene expression signature. In contrast, in the presence of EC, scRNAseq cell profiling demonstrated that 5.9% of fibroblasts were found to transcriptionally cluster with *THY1^+^* perivascular fibroblasts whereas 15.4% of fibroblasts clustered in association with mural cells suggesting signals from the EC differentiate fibroblasts into both perivascular fibroblasts and mural cells. Furthermore, receptor ligand analysis showed EC induced *THY1^+^* fibroblast and mural cell differentiation through DLL4/JAG1:NOTCH3 signaling (86). The genetic deletion of *Notch3* or antibody mediated therapeutic blockade of NOTCH3 signaling led to attenuated arthritis (86).

Recently, Buechler et al. (2021) (123) demonstrated universal fibroblast subsets (*Pi16^+^* and *Col15a1^+^*) exist across all tissue types (including the joint). These subsets express markers of stemness and are thought to give rise to specialized fibroblast subsets (dependent on tissue type and state) where *Pi16^+^* transition through the *Col15a1^+^* intermediate state. These universal fibroblasts also expressed high amounts of dermatopontin (DPT) and genetically engineered mice which express the fluorescently labelled DPT protein confirmed their transcriptional trajectory giving rise to three specialized fibroblast subsets demarked by genes *Cxcl5*, *Adamdec* and *Lrrc15*. In RA synovial tissue, *Pi16^+^* fibroblasts were shown to give rise to *Cxcl5^+^* fibroblasts which expressed high levels of proinflammatory cytokines IL-6 ad IL-1β and chemokines CCL2 and CCL7 thought to be located in the sub-lining. Co-regulated gene analysis also confirmed this activated cluster was driven by Pi3K, TNF and NFκB signaling. In summary, universal Pi16^+^ fibroblasts are plastic and differentiate into specialized fibroblasts following a site-specific signal such as NOTCH signaling in an inflamed joint.

In agreement with the above studies, others have demonstrated similar pro-inflammatory specialized fibroblasts exist across multiple chronic inflammatory diseases: RA, inflammatory bowel disease (IBD), interstitial lung disease and Sjögren’s syndrome (124). Again, using scRNAseq, this group discovered two shared fibroblast clusters exist across these diseases: *SPARC3^+^ COL3A1^+^* and *CXCL10^+^ CCL19^+^*. The authors propose *SPARC3^+^ COL3A1^+^* fibroblasts contain a conserved developmental pathway which drive tissue remodelling in early stages of disease to support chronic inflammation, whereas *CXCL10^+^ CCL19^+^* fibroblasts are immune interacting and promote cellular infiltration. Thus, *SPARC3^+^ COL3A1^+^* cells interact with blood vessels and alter the EC matrix to promote or inhibit infiltration (during early or resolving arthritis) whereas *CXCL10^+^ CCL19^+^* fibroblast differentiation supports immune cell infiltration during active RA. In agreement with Wei et al. (2020) (86), the authors suggest that *SPARC3^+^ COL3A1^+^* perivascular fibroblasts cross-talk with EC through DLL4:NOTCH3 signalling as well as TGF-β1, PDGFβ and angiogenic and mitogenic factors ephrin-α and -β and MDK and PTN respectively.

Furthermore, the investigators demonstrate that *CXCL10^+^ CCL19^+^* synovial fibroblasts display a strong IFN*γ* response signature (124) and are therefore believed to interact directly with CD8+ T cells (IFN*γ* is abundantly expressed by CD8+ T cells). As a result, it is likely these immune-inflammatory fibroblasts are analogous to Zhang et al. (2019) *HLA-DR^High^* pathogenic fibroblasts (42). Furthermore, the expression of synovial fibroblast CX_3_CL1 and CX_3_CR1 expressed by CD8+ T cells has been shown to positively correlate with RA severity (125) and monoclonal antibody treatment targeting CX_3_CL1 has shown to be successful in early phase clinical trials in RA (126). This suggests, in conjunction with NOTCH signaling, infiltrating IFN*γ* producing CD8+ cells may also play a role in activating pathogenic fibroblast subsets such as *HLA-DR^High^*. It is highly likely other signals arising from resident and infiltrating cells will evolve during disease progression that ultimately lead to modulation of fibroblast phenotype.

In summary, synovial fibroblasts demonstrate significant plasticity in response to local signals from within the joint microenvironment and give rise to transcriptionally distinct phenotypes based on their anatomical location within the synovium. These positional cues are orchestrated to create distinct synovial tissue niches that support infiltrating inflammatory cells. Under inflammatory states, the expression of transcriptional cassettes encoding the pathogenic fibroblast phenotype appear to be transient and dependent on positional cues. However, in chronically inflamed joints, fibroblasts are exposed to persistent inflammatory cues and, therefore, these signatures may become epigenetically imprinted, developing a sustained pathogenic fibroblast phenotype (29). The role in which epigenetics plays in modulating fibroblasts phenotypes in RA will be discussed below.

## Pathological Fibroblast Cell States Drive the Persistence of Joint Inflammation

Historical evidence demonstrates fibroblasts from the RA synovium of patients with advanced disease, including those undergoing joint replacement, have an aggressive and invasive phenotype that is maintained when engrafted into a severe combined immunodefient mouse (SCID mice) (lacking B and T cells) (38, 127, 128). It is therefore hypothesized that epigenetic modifications underpin the persistently activated fibroblast cell state and the invasive phenotype observed in chronic disease.

Unlike genetic mutations, epigenetic modifications are reversible as they do not alter the genetic sequence, only the way the gene is transcribed. Thus, epigenetic modifications in the promotor region of genes suppress and exacerbate their expression. It is suggested there are three main types of epigenetic modifications reported in RA synovial fibroblasts: DNA methylation, histone modifications and micro RNA (miRNA) expression [reviewed in (29)]. Such modifications are disease specific showing different epigenetic profiles in RA compared to OA joint fibroblasts (129, 130). In addition, current investigations have now identified long non-coding RNAs (lncRNAs) as master regulators of epigenetic modifications in RA synovial fibroblasts [reviewed in (131)] which will also be discussed below.

DNA methylation is the covalent transfer of a methyl group to the 5’ position within CpG islands by DNA methyltransferase (DNMT). DNA methylation silences or suppresses gene expression whereas hypomethylation (unmethylated CpG site that is normally methylated) enhances expression. In RA, the fibroblast methylome state is predominantly hypomethylated and has been shown to promote the aggressive phenotype by enriching the expression of genes associated with immuno-inflammation, proliferation and apoptosis (132). Hypomethylation also generates destructive endogenously activated pathways in RA fibroblasts through the increased expression of CXCL12 which activates MMPs *via* CXCR7 (133).

Recent evidence suggests changes in DNA methylation occur early in RA (undifferentiated RA) and has prognostic potential (134). From this study the total number of hypermethylated CpG sites increased from 9% in undifferentiated arthritis to 84% in very early RA (symptom duration ≤3 months) and 96% established RA (≥3 months duration). Furthermore, specific hypermethylated CpG sites were observed in all stages of disease including downregulation of the *MFAP2* gene in established RA. These findings suggest, in established RA, fibroblast hypermethylation may cause a reduction in *MFAP2* expression by *DKK3^+^* immune-regulatory fibroblasts observed by Zhang et al. (2019) (42).

Inhibiting histone acetylation using various histone deacetlylase (HDAC) inhibitors attenuates experimental arthritis in mice (135, 136). Also, prolonged exposure to inflammatory mediators such as TNFα loosens chromatin accessibility in RA synovial fibroblasts by decreasing total histone-4 (H4) and hyperacetylating the remaining (137). Such modifications prime fibroblasts and enhance transcription of NFκB and production of proinflammatory cytokines and chemokines such as IL-6 and CXCL10, CXCL9 and CXCL11 respectively. Reports also suggest synovial fibroblast histones can be methylated which can occur on lysine and arginine residues only (with prevalence for lysine) and is regulated by histone lysine methyltransferase (HKMT) and demethylases (HKDM) (138). In response to TNFα stimulation, HKM-modifying enzymes dysregulate HKMT and HKDM activity resulting in changes in synovial fibroblast gene expression.

The production of miRNAs by synovial fibroblasts also alters their gene expression – a phenomenon driven by proinflammatory mediators which are present in the inflamed joint microenvironment. The RA synovial fibroblast miRNA signature determines the aggressive phenotype of synovial fibroblasts observed in chronic arthritis where specific miRNAs have been shown to target mechanisms that regulate methylation (139). Many synovial fibroblast miRNAs have been identified in RA synovial fibroblasts which exhibit enhanced or suppressive effects on proinflammatory gene transcription (140–142). In contrast, one study suggests miRNA expression can result in a pro-resolving state, for example resolvin D1 treatment in CIA in mice suppressed angiogenesis, pannus formation (determined by synovial expression of connective tissue growth factor) and proinflammatory cytokine levels through the upregulation of miRNA-146a-5p expression which inhibited STAT3 activation (143). So, it appears that expression of miRNAs by synovial fibroblasts has direct pathological, protective and pro-resolving outcomes which become dysregulated in chronic inflammation.

Recent findings suggest lncRNAs are also important epigenetic regulators, and in RA these non-coding RNAs are not only reduced (144) but have both suppressive and stimulatory effects on synovial fibroblast gene expression, which ultimately supports chronic inflammation by enhancing fibroblast pro-inflammatory cytokine and chemokine production, proliferation, migration and invasiveness whilst preventing apoptosis (145). Others have shown lncRNA profiles differ in normal and RA patient plasma (146). In this study, RA patient samples contained a total of 169 upregulated lncRNA and 120 downregulated compared to healthy controls, suggesting changes in lncRNA fibroblast transcription is a biomarker of RA. It is also proposed lncRNAs can regulate fibroblast miRNA expression and therefore determine RA pathogenesis (147). For example, normal expression of fibroblast lncRNA *OIP5-AS1* is protective and inhibits the expression of miR-448 (148). In contrast, dysfunctional *OIP5-AS1* expression causes upregulation of miR-448 which induces TLR3/NFκB signalling and supported pathogenesis in arthritic rats. The exact mechanism lncRNAs play in RA is still yet to be determined, however Yan et al. (2019) suggest lncRNA *HIX003209* promotes a TLR4/NFκB proinflammatory phenotype in CD14+ infiltrating macrophages in RA by sponging miR−6089 (149).

While a persistent inflammatory stimulus can induce a sustained activatory phenotype in synovial fibroblasts, tissue resident macrophages appear to respond transiently and unlike fibroblasts maintain expression of immune-regulatory proteins such as ABIN3, IRAK-M, SOCS3 and ATF3 (150). Therefore, low expression of these immune-regulatory proteins in fibroblasts determines sustained activation. A more recent study demonstrates that 280 TNFα-inducible genes are repressed in macrophages following transient activation whereas fibroblasts sustain enhanced expression of 80 of these genes (151). These 80 genes also contained hyperacetylation of H3K27 and increased chromatin accessibility in NFκB promotor, interferon regulatory factors and activating protein-1. In summary, prolonged exposure to inflammatory stimuli causes epigenetic modifications in synovial fibroblasts which promote an aggressively imprinted proinflammatory phenotype. In contrast, TNFα exposure in macrophages induces a transient response which is tolerized. In RA patients who achieve clinical remission, it is possible macrophages may rapidly alter their response and transition to a homeostatic phenotype which supports resolution of inflammation and tissue repair. However, if fibroblasts maintain a sustained pro-inflammatory phenotype, they may fail to respond to the negative suppression by tissue resident macrophages, but this is yet to be proven. Further work is required to understand how epigenetically modified fibroblasts can be targeted in RA.

## Targeting Pathogenic Fibroblasts in Refractory RA

Targeting fibroblasts directly remains an aspirational goal but one that offers the potential to be less immunosuppressive than conventional biological therapies. One approach has been to target specific fibroblast cell behavior directly such as proliferation or hypertrophy/organization. A recent multi-center study termed TRAFIC (targeting synovial fibroblast proliferation in rheumatoid arthritis) conducted a phase 1b/2a clinical trial to determine the tolerated dose of seliciclib (152) [a cyclin-dependent kinase (CDK) inhibitor which induces expression of *p21* and suppresses expression of the pro-survival protein BCL-2] (153–156). Although the study deemed seliciclib as safe to continue forward into phase 2 trials, little is known about its therapeutic efficacy in RA currently. Similarly, the finding that *Cdh11^-/-^* mice exhibit a 50% reduction in clinical arthritis in mice (112) led to testing its therapeutic potential in RA. However, a phase I trial of a monoclonal antibody directed at CDH11 (RG6125) inflammation was completed but a phase II trial was discontinued owing to a lack of efficacy (126).

Strategies designed to directly target specific pathogenic fibroblast subsets are now being explored. Using a diphtheria toxin system in mice, we have recently shown that the selective deletion of FAPα expressing fibroblasts during inflammatory arthritis results in attenuation of synovial inflammation and reduced joint damage (39). These observations provide the rationale for therapeutic targeting of such pathogenic fibroblasts in RA. To translate these findings in humans it is important to identify such pathogenic subsets in the inflamed RA synovium and be able to effectively target these cells accurately while minimizing potential ‘off target’ effects. One possible approach to target FAPα+ synovial fibroblasts is the use of chimeric antigen receptor (CAR)-T cells. Local delivery of FAPα targeting CAR T cells in patients with pleural mesothelioma has been shown to be safe in a phase I clinical trial (157). Targeting FAPα expressing fibroblasts with CAR T cells following cardiac injury in mice has been shown to reduce cardiac fibrosis and restore cardiac function (158). However, further preclinical work is needed to determine the efficacy and specificity of FAPα CAR T cells in experimental arthritis.

Another potential therapeutic strategy is to inhibit local signals from within the joint which drive fibroblast activation states. For example, endothelium-derived NOTCH3 signaling inducing a pathogenic sub-lining phenotype in perivascular and interstitial fibroblasts. Genetic deletion or blockade of *Notch3* signaling has been shown to inhibit THY1^+^ sub-lining perivascular fibroblast expansion in mice and importantly attenuate experimental arthritis (86). NOTCH3 blockade has also been shown to reduce *SPARC3^+^ COL3A1^+^* fibroblasts but not *CXCL10^+^ CCL19^+^* fibroblasts (124). As it is hypothesized *SPARC3^+^ COL3A1^+^* fibroblast remodel the synovial vasculature to facilitate immune cell infiltration in early disease, it is probable NOTCH3 therapy could be more effective when employed in undifferentiated or very early RA cohorts.

In chronic disease, the prolonged exposure to proinflammatory mediators causes epigenetic histone modifications in synovial fibroblasts resulting in sustained expression of TNFα inducible genes (150, 151) – a phenomenon not observed in synovial macrophages (151). As this effect is due to histone hyperacetylation, targeting histone acetylation reader proteins such as BET bromodomain proteins could modulate the sustained or persistently activated fibroblast state. In support of this, treating synovial fibroblasts with the BET inhibitor I-BET151 significantly reduces proliferation, cytokine and MMP production (159). Furthermore, the effects of I-BET151 were shown to be cell-type specific inducing anti-inflammatory effects in macrophages. As mentioned above, histone acetylation primes fibroblasts enhancing expression of proinflammatory mediators. As histone acetylation is reversible it can be deacetylated by treating with histone deacetylases (HDAC). One study demonstrates that treatment of RA synovial fibroblasts with HDAC3 significantly reduces production of inflammatory mediators such as IFNs (160). This suggests potential for using HDACs to therapeutically target synovial fibroblasts in RA. Further work is required to identify and target critical epigenetic modifications and gene regulatory pathways that sustain pathogenic fibroblast states in inflammatory joint disease.

## Summary

During inflammatory arthritis, the spatial and temporal cellular heterogeneity of synovial macrophages and fibroblasts sees the emergence of specialized cellular subsets that define specilised synovial tissue niches that support the pathpogenic behavior of immune cells.

Early, inflammation driven remodeling of the synovial microenvironment is associated with a disrupted macrophage lining barrier that permits the invasion of activated fibroblasts and infiltrating immune cells. The expansion of pathogenic fibroblasts and alarmin-producing MERTK^-^ macrophages in the sub-lining layer results in a pro-inflammatory microenvironment that supports the infiltration and survivial of inflammatory cells. In order for inflammation to resolve, fibroblast-derived GAS6 promotes the differentiation of MERTK^+^ macrophages which in turn induce a reparative phenotype in lining fibroblasts. At this point the lining barrier function is restored and joint homeostasis is reinstated.

We propose that these pathways are associated with critical cellular checkpoints between macropahges and fibroblasts, and the failure of these bi-directional cellular interactions to suppress pro-inflammatory pathways results in the emergence of persistently activated pathogenic cell states that drive chronic inflammation. The failure of pathogenic fibroblasts to respond to negative regulation, such as those instructive signals provided by pro-resolving macropahges may underpin chronic inflammation and treatment refractory arthritis.

Future research strategies should combine the knowledge of cellular heterogeneity scRNAseq and *in vivo* and/or *in vitro* functional assays to understand the exact biological function of tissue resident synovial cells at different stages of disease. Furthermore, employing epigenetic profiling technologies such as open chromatin sequencing will assist in deciphering the epigenetic landscape of synovial fibroblasts and identify differences in gene regulation that lead to specific programs of gene expression that underpin their phenotypic heterogeneity and how these are modulated during chronic inflammation. Such findings will enable the identification of novel fibroblast targeted therapies designed to break the ceiling of therapeutic response in RA.

## Author Contributions

Both authors contributed equally to the review. All authors contributed to the article and approved the submitted version.

## Funding

AC is funded by a Kennedy Trust for Rheumatology Research Senior Clinical Research Fellowship KENN 192006.

## Conflict of Interest

The authors declare that the research was conducted in the absence of any commercial or financial relationships that could be construed as a potential conflict of interest.

## Publisher’s Note

All claims expressed in this article are solely those of the authors and do not necessarily represent those of their affiliated organizations, or those of the publisher, the editors and the reviewers. Any product that may be evaluated in this article, or claim that may be made by its manufacturer, is not guaranteed or endorsed by the publisher.
